# Parrots have evolved a primate-like telencephalic-midbrain-cerebellar circuit

**DOI:** 10.1038/s41598-018-28301-4

**Published:** 2018-07-02

**Authors:** Cristián Gutiérrez-Ibáñez, Andrew N. Iwaniuk, Douglas R. Wylie

**Affiliations:** 1 Neuroscience and Mental Health Institute, University of Alberta, Edmonton, AB T6G 2E9 Canada; 2 Department of Neuroscience, Canadian Centre for Behavioural Neuroscience, University of Lethbridge, Lethbridge, AB T1K 3M4 Canada

## Abstract

It is widely accepted that parrots show remarkable cognitive abilities. In mammals, the evolution of complex cognitive abilities is associated with increases in the size of the telencephalon and cerebellum as well as the pontine nuclei, which connect these two regions. Parrots have relatively large telencephalons that rival those of primates, but whether there are also evolutionary changes in their telencephalon-cerebellar relay nuclei is unknown. Like mammals, birds have two brainstem pontine nuclei that project to the cerebellum and receive projections from the telencephalon. Unlike mammals, birds also have a pretectal nucleus that connects the telencephalon with the cerebellum: the medial spiriform nucleus (SpM). We found that SpM, but not the pontine nuclei, is greatly enlarged in parrots and its relative size significantly correlated with the relative size of the telencephalon across all birds. This suggests that the telencephalon-SpM-cerebellar pathway of birds may play an analogous role to cortico-ponto-cerebellar pathways of mammals in controlling fine motor skills and complex cognitive processes. We conclude that SpM is key to understanding the role of telencephalon-cerebellar pathways in the evolution of complex cognitive abilities in birds.

## Introduction

**Figure 1:**
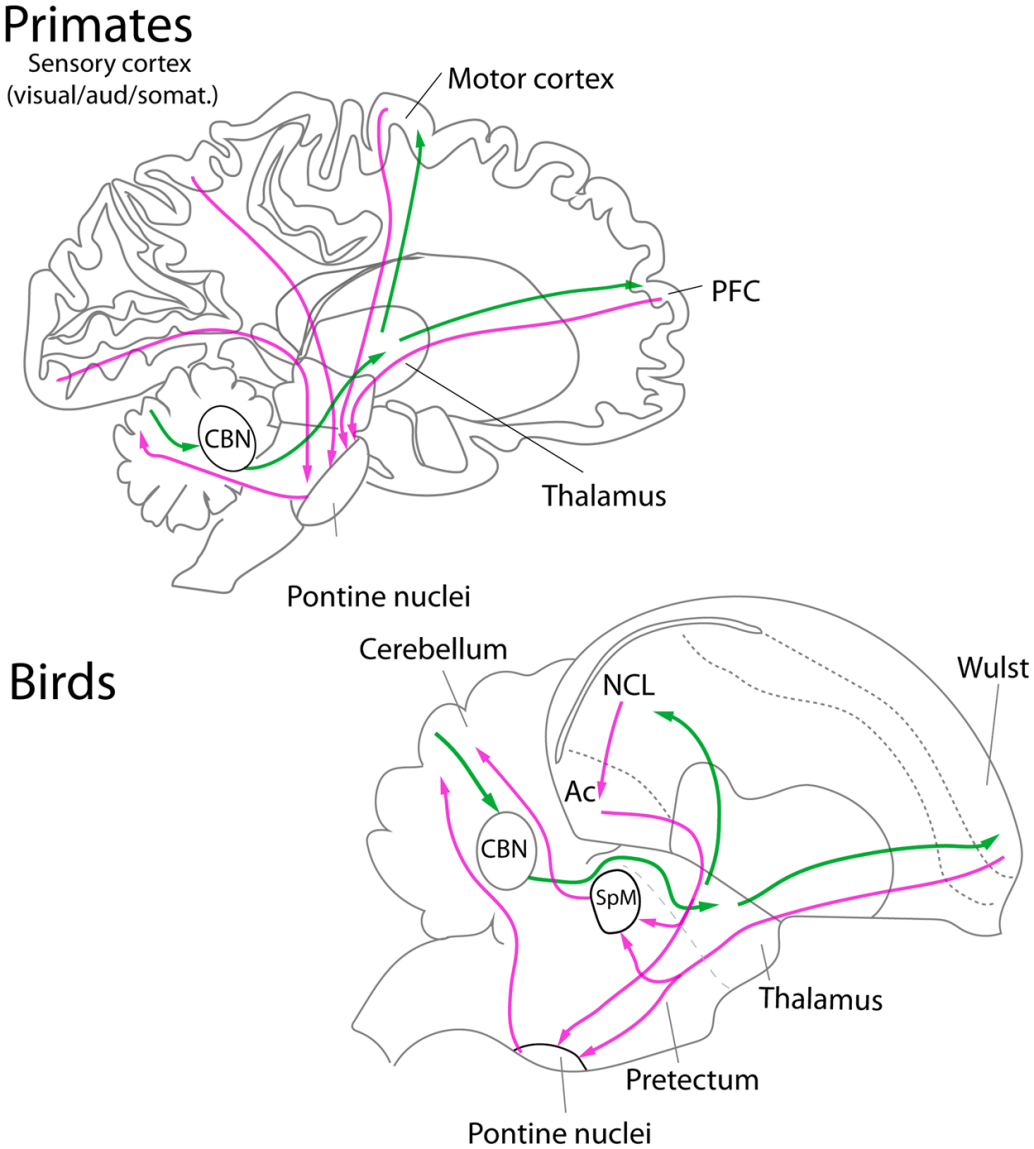
Cortico-cerebellar pathways in birds and mammals. In mammals, inputs from the cortex to the cerebellum are routed through the pontine nuclei. In birds, inputs from the telencephalon to the cerebellum are also routed through two nuclei in the base of the pons (medial and lateral pontine nuclei, PM and PL) but also trough an additional nucleus in the pretectum, the medial spiriform nuclei (SpM). In mammals the cerebellum, through the cerebellar nuclei (CBN) sends projections back to the thalamus, which in turn projects to motor and associative areas of the cortex. In birds there also a projection from the cerebellum to the thalamus but this arises from the lateral cerebellar nucleus. In turn, these regions of the thalamus projects the nidopallium caudolaterale (NCL), (the avian analogous of the prefrontal cortex of mammals), and the rostral Wulst, (the avian equivalent of motor cortex).

It is widely accepted that parrots show complex cognitive abilities. These include: tool manufacture, mirror self-recognition, object permanence, meta-cognition, theory of mind, vocal learning, mental time travel and complex social cognition^1–3^. This array of cognitive behaviors is only matched by corvids and primates reviewed in^2,3^. In birds and mammals the convergent evolution of these cognitive abilities has been associated with convergent changes in the brain, including an increase in the size of the whole cortex/telencephalon^4–6^ and/or specific parts of it, such as the prefrontal cortex (PFC) in mammals e.g.^7^ or the associative areas of the pallium in birds e.g.^8^. In recent years, it has been increasingly recognized that complex cognitive abilities (or any behavior) cannot be assigned to a single brain structure; rather they arise from distributed neural systems^9^. In the case of primates, increasing evidence suggests that the cerebellum and cortico-cerebellar pathways play an essential role in complex cognitive abilities like the ones mentioned above^10,11^. Accordingly, the expansion of the cortex in primates has been accompanied by an increase in the size of the cerebellum and different components of cortico-cerebellar pathways^9,12^, in particular, the circuit between the PFC and cerebellum that includes the pontine nuclei and parts of the thalamus (Fig. 1)^9,12–15^.

Parrots have a relatively large telencephalon, similar in size to primates^16,17^, and as in primates, this expansion of the telencephalon is thought to reflect cognitive abilities. Although birds and mammals share many similarities in brain structure, the cortico-cerebellar pathways differ markedly between the two clades. In primates, one of the largest circuits in the brain originates in the cortex and projects to the cerebellum through the pontine nuclei^18^. The cerebellum then provides feedback to the cortex through projections of the cerebellar nuclei to the thalamus^10^ (Fig. 1). In contrast, a cortico-ponto-cerebellar system appears to be poorly developed in birds^19,20^. Birds have projections from the telencephalon similar to the mammalian pyramidal tract, but this too is poorly developed^21–23^. There are two proposed homologs to the pontine nuclei of mammals in the brainstem of birds, the lateral and medial pontine nuclei (PL and PM respectively; 19), which send crossed projections to lobules VI–IXb of the cerebellum^19^ and receive projections from the telencephalon^21–23^. Additionally, the pontine nuclei of birds receive direct projections from several retinorecipient nuclei including the optic tectum, the nucleus lentiformis mesencephali, the accessory optic system and the ventral thalamus Reviewed in^24^. Birds also have a third nucleus that is analogous to the pontine nuclei^20^ as it receives projections from the telencephalon and sends projections to the cerebellum: the medial spiriform nucleus (SpM)^20–23^. Although Finger and Karten^20^ ascribed SpM as a thalamic nucleus, developmental studies suggest that SpM is pretectal in origin^25^. If the evolution of complex cognitive abilities are dependent upon enlargements of a cortico-ponto-cerebellar pathway^9,12–15^, then parrots should have enlarged pontine and/or SpM nuclei, and these nuclei will covary with the size of their afferent and efferent targets. In this paper, we test these hypotheses in a large, comparative dataset and show that SpM is not only enlarged in parrots, but that only the SpM nucleus covaries with telencephalon size in birds, adding further evidence to the convergent evolution of brain structure and cognition in parrots and primates.

## Results

**Figure 2:**
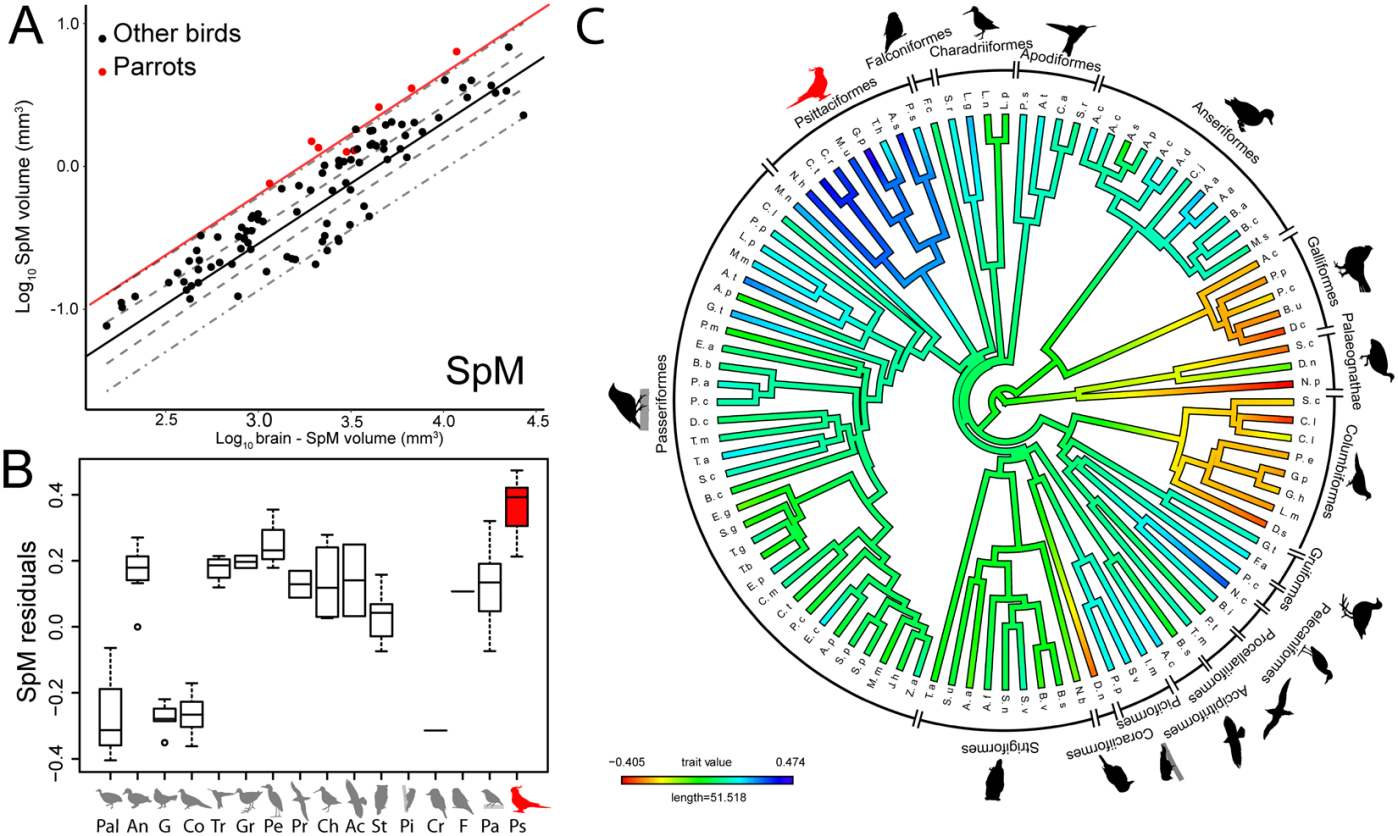
Relative size of the medial spiriform nucleus (SpM). (**A**) The log-transformed volume of the SpM is plotted as a function of the brain volume minus the volume of SpM. Black solid line represents the regression line for all species obtained from a PGLS (see methods). The red line shows the regression line for parrots also obtained from a PGLS. Confidence intervals are presented as dashed lines, prediction intervals as dash and dotted lines in grey. (**B**) Box plots show the relative size of SpM for each avian orders represented in this study. Parrots (Psittaciformes), are shown in red. Values shown in the box plots are derived from the residuals from PGLS of the log of the volume of each nucleus against the brain volume (see methods, panel A). The solid horizontal line represents the median, the two boxes represent the limits of the second and third quartiles and the whiskers (dashed vertical lines) represent the limits of the first and fourth quartiles. The open circles represent outliers, which are defined as those outside the first or fourth quartile by a magnitude of 1.5X the inter-quartile range. (**C**) Ancestral character estimates for relative SpM volumes plotted onto an avian phylogeny. This method uses ancestral character estimation to visualize historical character states for SpM volumes (plotted as a continuous trait) along the branches of a tree. The trait mapped was the relative size of SpM as in panel B. An = Anseriformes, Ch = Charadriiformes, Co = Columbiformes, F = Falconiformes, G = Galliformes, Gr = Gruiformes, Pa = Passeriformes, Pal = Paleognaths, Pe = Pelecaniformes, Pi = Piciformes, Pr = Procellariiformes, Ps = Psittaciformes, St = Strigiformes, Tr = Trochiliformes.

**Figure 3:**
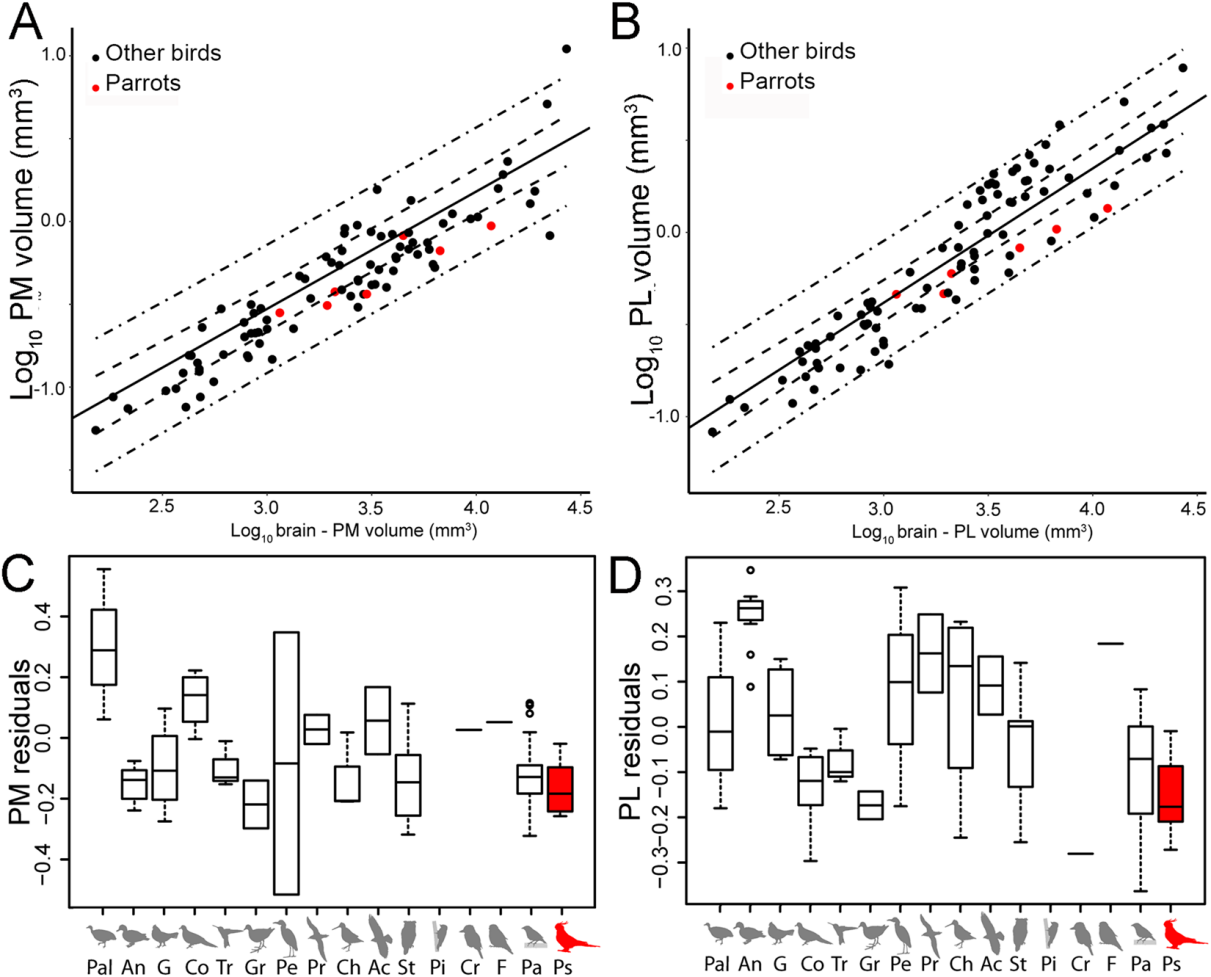
Relative size of the medial and lateral pontine nuclei (PM, PL). (**A**,**B**) The log-transformed volume of the PM and PL is plotted as a function of the brain volume minus the volume of each structure (PM = A; PL = B) for all species examined (see methods and supplementary materials, Table S1). Parrots (Psittaciformes) are shown in red. (**C**,**D**) Box plots show the relative size of PM (**C**) and PL (**D**) for each avian orders represented in this study. Parrots (Psittaciformes), are shown in red. Values shown in the box plots are derived from the residuals from PGLS of the log of the volume of each nucleus against the brain volume (see methods, panel A,B). The solid horizontal line represents the median, the two boxes represent the limits of the second and third quartiles and the whiskers (dashed vertical lines) represent the limits of the first and fourth quartiles. The open circles represent outliers, which are defined as those outside the first or fourth quartile by a magnitude of 1.5X the inter-quartile range. An = Anseriformes, Ch = Charadriiformes, Co = Columbiformes, F = Falconiformes, G = Galliformes, Gr = Gruiformes, Pa = Passeriformes, Pal = Paleognaths, Pe = Pelecaniformes, Pi = Piciformes, Pr = Procellariiformes, Ps = Psittaciformes, St = Strigiformes, Tr = Trochiliformes.

Figure 2 shows that SpM is significantly enlarged in parrots compared with other birds, (see also Table S3). The enlargement of SpM is a ‘grade shift’ based on our analyses (Table S3); SpM in parrots does not change in size at a different rate with increasing brain size than other birds, but is rather an increase in the size of SpM relative to brain size (Fig. 2A,B: no difference in slopes, F_1,87_ = 0.0482, p = 0.8268). The significant difference in the size of SpM was unaffected by phylogenetic uncertainty (Fig. S1) or influential cases (see methods, data not shown). Figure 2C illustrates the relative size of SpM mapped across a phylogenetic tree of all species measured. Compared with other major avian groups (e.g., songbirds, waterfowl, owls), only parrots have universally large relative SpM volumes, indicating that this is a characteristic feature of parrots (Fig. 2B,C). This was confirmed with pANCOVAS that tested if there are grade shifts in the size of SpM in other orders that have positive SpM residuals (Anseriformes, Strigiformes, Passerifomes and Pelecaniformes). None of these groups differed significantly in the size of SpM when compared to all other birds (Table S3). Contrary to SpM, the two pontine nuclei, PM and PL, are not enlarged in parrots (Fig. 3 and Table S3).

We then compared the size of the telencephalon and cerebellum between parrots and other birds. A pANCOVA with brain minus the telencephalon as the independent variable shows no significant difference in the size of the telencephalon between parrots and all other birds (F_1,73_ = 3.11, p = 0.082), which is similar to an earlier analysis^16^. This non-significant difference between parrot and other birds is likely driven by the large telencephalon of owls, which have undergone an enlargement of the hyperpallium associated with visual processing and not cognition^16,26,27^. When owls are removed, there is a significant difference in the relative size of the telencephalon between parrots and all other bird species that we were able to include in our dataset (ʎ = 0.90, F_1,76_ = 7.37, p > 0.001). When tested in a similar way, we found no significant difference in the size of the cerebellum between parrots and all other birds (ʎ = 0.85, F_1,73_ = 0.25, p = 0.615). Finally, we tested the difference in size of SpM, telencephalon and cerebellum between parrots and other birds in models with the brain volume minus SpM, telencephalon, optic tectum and cerebellum volume as an independent variable. The optic tectum was included as a proxy for the size of visual structures in different species, as its relative size is correlated with that of other visual structures in birds (see methods). In this case pANCOVAs reveal a significant difference in the size of SpM (ʎ = 0.89, F_1,73_ = 4.09, p < 0.05) but not the telencephalon (ʎ = 0.94, F_1,73_ = 1.50, p = 0.22) and cerebellum (ʎ = 0.95, F_1,73_ = 0.21, p = 0.64) between parrots and all other birds.

We then used PGLS models to test wether the relative size of SpM is correlated with that of the telencephalon, cerebellum and optic tectum in birds in general (Table 1). Our full model shows a strong and significant effect of telencephalon on the size of SpM, but not one of the cerebellum or the optic tectum. Given that we found high collinearity between our variables and this can potentially be problematic in estimating the effect of each variable (see methods, Table 1), we tested for the stability of our full model in two ways (see methods for details). First, removing data points one by one did not change the sign of the effect of each variable on the size of SpM and only moderately changed the size of the estimated coefficient (range 0.764 to 0.818 for Tel; 0.104 to 0.184 for Cb and −0.175 to −0.075 for TeO). Second, removing the cerebellum or optic tectum from the model did not change the sign any of the coefficient estimates (Table 1). Coefficient estimates did change, but in a predictable manner given the collinearity between variables. When two collinear predictors have similar qualitative effects and positive correlations, like in the case of the telencephalon and cerebellum on SpM, removing one will overestimate the effects of the other^28,29^. Our full model shows that the highest collinearity (see methods) is between the cerebellum and the telencephalon (Table 1), and as expected, removing either of them results in a higher value of the estimate of the other, particularly the cerebellum, and a reduced error of the estimate (Table 1). When the telencephalon was not included (reduced models 3 and 6) there was a significant and positive effect of the relative size of the cerebellum and SpM. The predicted consequences of collinearity are: (a) the aforementioned overestimation of the effect of one variable in the absence of its collinear variable^28,29^, (b) the overestimation of the effect of the collinear variable with the largest effect, and underestimation of that with the weakest; and c) the increase in error in the estimate effect and therefore increased likeness to find non-significant effects^30^. Based upon these effects of collinearity, our data suggest that there is a strong correlation of the relative size of SpM with the telencephalon and a weaker, or even non-significant, correlation between SpM and the cerebellum, but these effects cannot be effectively separated.

**Table 1:** Results of PGLS models with SpM as the dependent variable.

Model	ʎ	AIC	Variable	VIF*	β	S.E	t	P value
Full	0.94	−126.36	RBV	5.01	−0.02	0.061	−0.33	0.7458
			TeL	12.21	0.79	0.126	6.29	<0.0001
			Cb	15.05	0.14	0.121	1.12	0.26
			TeO	6.50	−0.15	0.103	−1.44	0.15
Reduced 2	0.96	−126.34	RBV	4.91	−0.02	0.061	−0.44	0.65
			TeL	12.61	0.74	0.123	6.08	<0.0001
			Cb	11.58	0.08	0.114	0.75	0.45
Reduced 3	0.94	−127.05	RBV	4.89	−0.03	0.059	−0.56	0.58
			TeL	6.88	0.89	0.088	10.15	<0.0001
			TeO	5.01	−0.11	0.097	−1.15	0.25
Reduced 4	0.96	−94.34	RBV	4.51	0.17	0.063	2.75	0.007
—			Cb	8.48	0.67	0.102	6.59	<0.0001
			TeO	6.48	0.01	0.121	0.11	0.91
Reduced 5	0.95	−127.76	RBV	4.06	−0.03	0.059	−0.59	0.55
			TeL	4.06	0.82	0.063	12.92	<0.0001
Reduced 6	0.96	−96.32	RBV	4.38	0.17	0.061	2.88	0.005
			Cb	4.38	0.68	0.071	9.49	<0.0001

RBV is the remaining brain volume, brain volume minus telencephalon (Tel.), cerebellum (Cb) and optic tectum (TeO).

*Calculated from a non-phylogenetic model, see methods.

We performed similar analysis for PM and PL (Tables S4 and S5). In this case, we included the size of visual nuclei that send projections to each pontine nuclei (the ventral geniculate nucleus (Glv) and the nucleus lentiformis mesencephalic (LM) in the case of PM, and the optic tectum and the nucleus of the basal optic root (nBOR) in the case of PL, see methods). The relative size of both PM and PL is correlated with that of the telencephalon and the cerebellum, although collinearity makes it difficult to discern the exact effects between telencephalon and cerebellum and the size of PM or PL. The relative size of PM is also correlated with relative size of Glv and/or LM, while PL was not significantly correlated with any visual nuclei. Finally, we also tested if there is a correlation between the size of the telencephalon and the cerebellum in birds. A pgls of telencephalon volume regressed on volume of cerebellum controlling for volume of the rest of the brain suggest a correlation of the size of the telencephalon and the cerebellum in birds (ʎ = 0.93, t_2,73_ = 13.13, p = >0.00001).

In summary, our results show: (1) SpM and the telencephalon but not the pontine nuclei or the cerebellum, is relatively large in parrots compared to other birds (Figs 2, 3 and Table S3); and (2) the relative size of SpM is correlated with that of the telencephalon across all birds (Table 1).

## Discussion

Parrots and primates show impressive convergence of complex cognitive abilities^1,2^ and this is accompanied by convergent changes in the brain, such as: relatively large brains^6,16,17^, relatively large telencephalons^26^ and particularly large associative areas of the telencephalon^31^. Our results show an additional convergence between these two groups. We found that SpM, a nucleus that acts as a relay between the forebrain and the cerebellum, is particularly large in parrots compared to other birds (Table S3 and Fig. 2), and that the relative size of SpM is strongly correlated with the relative size of the telencephalon across all birds (Table 1). While this parallels the situation in primates, in so far that the size of the cortex is strongly correlated with that of the pons^9,15^, our results suggest, at most, a weak correlation between the relative size of SpM and the cerebellum. Nonetheless, this accounts for an impressive case of convergent evolution because SpM is not part of the pons, but rather located in the dorsal part of the border between the thalamus and pretectum^20,25^. Mammals do not have a comparable nucleus and among vertebrates, only teleosts have similar thalamic/pretectal nuclei that connect the forebrain with the cerebellum^20,32^. Our results also show that while the relative size of the pontine nuclei show some correlation with that of the cerebellum, these nuclei are not enlarged in parrots, (Tables S4 and S5). Thus, parrots and primates have convergently evolved increased connectivity between the telencephalon and the cerebellum, but have done so through different neural pathways.

The similarities in the evolution of cortico-cerebellar pathways between parrots and primates is not at all levels. While primates have a relative large cerebellum when compared to other mammals^11^, previous studies and our results show that parrots do not have a larger cerebellum than other birds (see results^16^). Despite the lack of an enlarged cerebellum in parrots, we did find a correlation between the relative size of the telencephalon and the cerebellum across all birds (see results), which seems to mimic the situation in primates and mammals^9,11^. However, this result should be interpreted with caution. In this study we compared the size of the whole telencephalon with that of the cerebellum while in mammals the comparison has been between the cortex and the cerebellum. It is possible that the inclusion of the pallium and sub-pallium in our data has affected our results, and that the relative size of the pallium is not correlated to that of the cerebellum in birds. Interestingly, parrots do show some differences in the cerebellum when compared to other birds. Previous research has shown that, relative to their brain size, parrots have more folded cerebella when compared to other birds^33^. It is therefore possible that the relative size of SpM is correlated to other aspect of cerebellar anatomy, such as the number or size of Purkinje cells.

**Figure 4:**
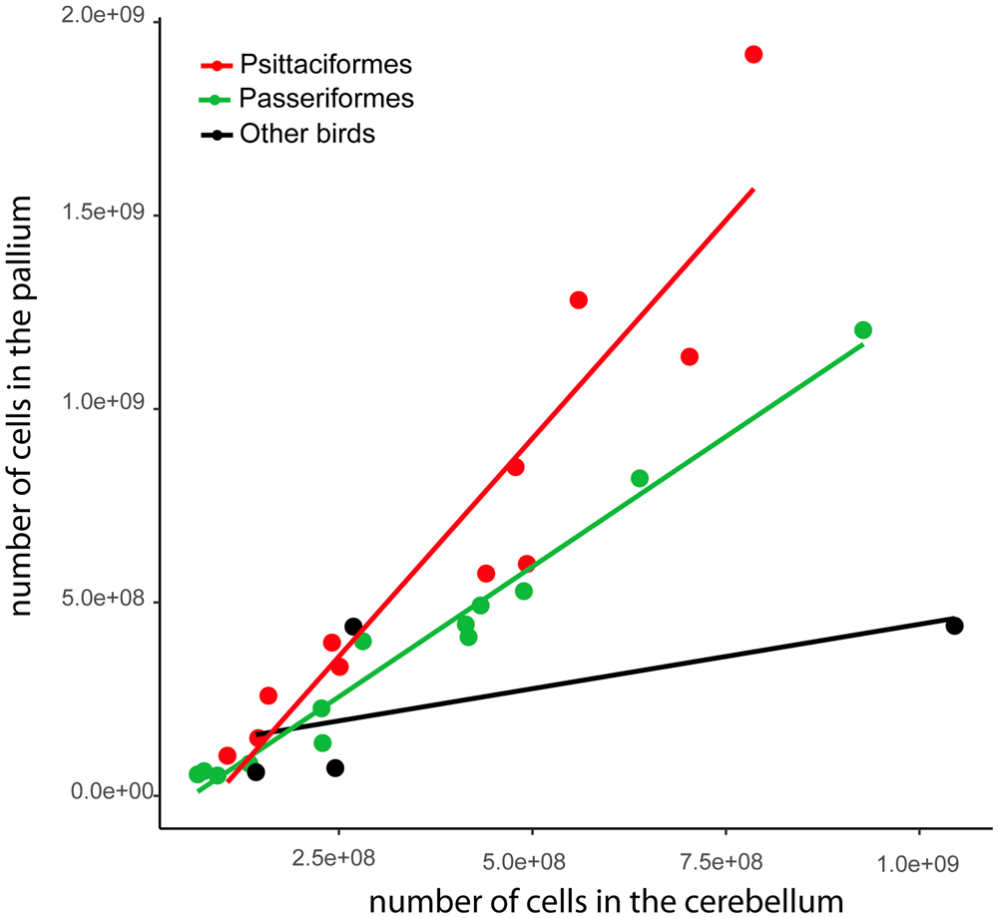
Scatterplot of the number of cells in the cerebellum of several species of birds versus the number of neurons in the pallium. Data from^36^.

The differences in the evolution of cortico-cerebellar pathways, particularly the cerebellum, between parrots and primates is not completely surprising given several major differences in the cerebellum of birds and mammals. The most prominent is the absence of a clear homologues of the cerebellar hemispheres in birds and their main output, the dentate nucleus^34^. This may be particularly relevant to the evolution of cortico-cerebellar pathways in birds, given that the main output of the dentate nucleus is regions of the thalamus that then projects to the cortex^10^, and these are precisely the components of the cerebellum that are enlarged in primates^14,15^. Also, while the cerebellum of primates contains up to 80% of the total number of cells in the brain^35^, in parrots the cerebellum only contains 40% or less of the total number of neurons in the brain, which results in a much lower ratio of cerebellar to pallial neurons in birds than mammals^36^. Further, the ratio between pallial and cerebellar cells is constant across all mammals, but varies among avian groups (see below, Fig. 4). This suggests that even though there are compelling convergences between birds and mammals in brain morphology and behavior, there are also some fundamental differences in how their brains are constructed. The implications of these differences for cognition are as yet unknown.

While we did not find an expansion of the cerebellum or the pontine nuclei in parrots, our results suggest that across all birds exists a correlation between the size of these structures and the telencephalon. The correlation between the cerebellum and the telencephalon does not only mimics that found in primates (see above), but also in sharks^37^, which suggest that a correlated evolution of this two structures maybe wide spread among vertebrates. Additionally, the concerted evolution of the size of these two structures with that the pontine nuclei and SpM is similar to the correlated evolution of functional circuits in other systems of the avian brain, including the tectofugal pathway, other sensory pathways, and the song system^38–41^, as well to the correlated evolution of functional circuits in the brain of mammals^9,11^. This further supports the notion that interconnected structures evolve together and that neural circuits should be taken in consideration when studying the evolution of the brain.

### Correlation between SpM and telencephalon

In primates, the expansion of the neocortex has been accompanied by the enlargement of existing areas and addition of new ones, in both primary sensory areas, mostly visual, and also sensorimotor, premotor and motor areas^42,43^. Sayol *et al*.^31^ has recently shown that the expansion of the telencephalon in parrots and corvids is mostly explained by an expansion of the associative pallium (AP), which they defined as the nidopallium and mesopallium plus its main output, the arcopallium. The AP contains both primary sensory circuits (visual, auditory, trigeminal-somatosensory), as well as premotor/motor and associative circuits or areas (Reviewed in^44^). In this sense, the expansion of the AP in parrots could resemble the expansion of the cortex in primates more closely than that of other clades of birds, like owls and waterfowl, where the expansion of the telencephalon is driven by the expansion of primary sensory areas^26,27,45,46^. It is currently not known if the expansion of the AP in parrots and corvids is related to particular circuits or all of them, but there is little evidence that parrots or songbirds have expanded visual, auditory and/or somatosensory areas of the AP^39,46,47^, suggesting that the expansion of the AP in parrots and corvids may be more associated with the expansion of associative areas, like the caudolateral nidopallium (NCL).

The concomitant expansion of SpM and the telencephalon in parrots suggest that like in mammals, the evolution of complex cognitive abilities is linked with an increase in the connectivity between the telencephalon and cerebellum. As mentioned before (see introduction), it is widely recognized in mammals that the circuits that connect the telencephalon with the cerebellum are important for complex cognitive abilities, but in birds all attention so far has concentrated almost exclusively on the telencephalon and no consideration has given to how extra-telencephalic circuits may contribute to the cognitive abilities of parrots and other birds.

In birds most studies focus on the NCL as the substrate for complex cognitive abilities^3,48^. NCL is considered the closest avian equivalent of the mammalian prefrontal cortex, based on hodological, electrophysiological, functional, and neurochemical evidence (reviewed in^49^). In primates, trans-synaptic tracing has shown direct connections between the prefrontal cortex and the cerebellum^10,50^ (Fig. 1). In birds, comparable trans-synaptic tracing has yet to be done, but traditional tracing studies strongly suggest that the AP, including NCL, connects with the cerebellum through SpM. As mentioned before, the arcopallium projects to SpM and receives projections from both the mesopallium and the nidopallium^44^. Wild and Farabaugh^51^ showed that in the zebra finch (*Taeniopygia guttata*), the lateral part of the intermediate arcopallium receives projections directly from the frontal part of the nidopallium and NCL, and this same area sends dense projections to the lateral SpM (Fig. 1). Interestingly, these same authors do not report any projections of this same area of the arcopallium to the PL, which other authors^21^ have reported from more general injections in the arcopallium of the pigeon. Thus, the projections from the arcopallium to SpM and PL arise from different populations of arcopallial cells and SpM appears to be the only relay between the NCL and the cerebellum. The lack of expansion of PL in parrots (Figs 3B,D and S1) supports this contention.

It is thought that the capacity of complex cognitive abilities in primates and some birds like parrots and corvids is related to the ability to generate mental representations of objects thus allowing the mental manipulation of these objects and the prediction of possible outcomes^52^. Through their extensive connections with sensory and premotor areas, the mammalian PFC and avian NCL are thought to perform executive control over these mental models in accordance with internal goals^53^. At the same time, the cerebellum encodes internal models that reproduce the crucial properties of mental representations in the cerebral cortex^10,54,55^. Then, connections from the PFC to the cerebellum allow the cerebellum to update these internal models if incongruencies are detected between the internal model and self-generated goals^52,55^. Our results showing a differential expansion of SpM in parrots suggest strongly that, as in mammals, the interaction of mental representations originated in the telencephalon with internal models from the cerebellum are also crucial for complex cognitive abilities in birds. However, this is accomplished through an alternative circuit that does not exists in mammals. It is not clear to what extent the NCL/Arcopallium-SpM-cerebellum pathway and the PFC-pons-cerebellum converge in terms of connectivity and physiology. In primates, and particularly humans, there is an expansion of the specific areas of the cerebellar hemispheres that receive projections from the PFC^14,56^. SpM sends projections from folia VI to VIII^20,57,58^, but these folia are not especially large in parrots^59^.

Another factor that is likely to contribute to the exceptional expansion of SpM in parrots, but not other birds, is the number and density of neurons. Relative to body size, parrots and songbirds have more neurons than other birds, and their neuronal densities exceed those found in mammals, especially in the pallium, the avian homologue of the cortex^36^. Although songbirds (including corvids) and parrots have similar neuronal numbers and densities in the pallium, there are also differences between these two groups. In examining the data provided in Olkowicz *et al*.^36^, we found that as the brain increases in absolute size, the pallium of parrots gains neurons with respect to the cerebellum at a higher rate than passerines and other birds (Fig. 4). In other words, parrots have a significantly higher ratio of pallial to cerebellar neurons than songbirds and other birds (parrots, 1.6: songbirds, 0.98: other birds, 0.7). Note that this is also different than mammals in two significant aspects. First, the ratio between pallial and cerebellar neurons in mammals is constant across groups; even though primates have much higher neuronal densities than other mammals^60^, the cerebellum and the pallium gain cells proportionate to each other across mammals. Second, this ratio is much lower in mammals than in birds (0.26 in mammals).

### Functions of SpM Expansion

The expansion of SpM that is unique to parrots (Fig. 2 and Table S3) may be key to the complex sensorimotor coordination and planning that is require for the extractive foraging that most species of parrots use for feeding. Most parrots feed on hard-shelled fruits and seeds. This requires a lot of fine manipulation to extract the nutritious contents from inside the fruit/seed. Usually, manipulation of these food items is achieved by coordination of a secure grasp of the objects with the foot, a muscular tongue and highly curved beak^61–63^. In mammals, several studies have highlighted the importance of sensorimotor cortico-cerebellar circuits in the fine control of voluntary movements e.g.^64^, but also  in sensorimotor prediction^65^. SpM is well suited for these tasks as, like the pontine nuclei of mammals, SpM receives projections from sensory, motor and premotor areas of the telencephalon of birds (Fig. 1). This includes the putative homologues of the visual, somatosensory and motor cortex of birds^22,23,66,67^ as well as the aforementioned connections with NCL^51,68,69^. The expansion of SpM may not only be crucial for the execution of complex foraging behavior of parrots but also the learning of complex motor skills. In mammals, cortico-cerebellar pathways are involved in the learning of complex motor behaviors, such as tool use e.g.^70^, and several studies suggest that parrots are only able to perform complex extractions after learning by observation of conspecifics^71,72^. The expansion of SpM may also be important for other behaviors which parrots are capable of and that demand the capacity for planning, execution and understanding complex behavioural sequences, such as the string pulling task^73^ and the use of tools e.g.^74^. Finally, it is possible that the enlargement of SpM in parrots is also important in the performance of complex social play behavior. Parrots are one of the few groups of birds that exhibit social play, along with corvids and hornbills^75^. Recently, Kerney *et al*.^76^, found a correlation between the size of the main components of cortico-cerebellar systems in primates and the amount of play behaviors exhibited. The expansion of SpM in parrots suggests the same could be true for birds.

### Conclusions and Considerations

Our results suggest that parrots’ brains may be unique among birds in having an expanded nucleus that connects the associative pallium with the cerebellum: the SpM. This suggests that, as in primates, telencephalon-cerebellar pathways play a major role in controlling complex behaviors. A major caveat in our understanding of the function of telencephalon-cerebellar pathways in birds is the lack of detailed information of the connections and organizations of these neural circuits in birds. For example, in mammals, an important component of cortico-cerebellar pathways are feedback projections from the cerebellum to the cortex via the cerebellar nuclei and the thalamus (see Fig. 1)^10^. This creates two loops between the cortex and the cerebellum: a motor loop and a prefrontal loop. It is unclear if birds (or other vertebrates) have homologs of the cerebellar hemispheres and the dentate nucleus, which gives rise to the cerebellar projections to the thalamus in mammals^34,77^. Instead, the lateral cerebellar nucleus of birds, which is thought to be homologous to the interposed nuclei of mammals^34^, sends projections to the dorsal thalamus, which then projects to either the rostral Wulst^67,78^, the putative homologue of the motor cortex in birds, or NCL (Fig. 1)^68^. This at least suggests the existence of both a motor and a prefrontal telencephalon-cerebellar loop in birds. Regardless of the specifics of these poorly understood pathways, our results clearly implicate SpM as a key brain region in the evolution of complex cognitive abilities in birds and provide a unique opportunity to reveal convergent characteristics of these circuits.

## Materials and Methods

### Ethics Statement

In all cases, the specimens were provided to us dead. Some of these species were collected dead from window strikes and culling operations in Australia by ANI under collection permits issued by the Victorian Department of Natural Resources and Environment. Other species were provided by other researchers, all of which had the correspondent capture/handling permits and/or ethics approval from their respective institutions. This includes Dr. Catherine Carr who had approval from the University of Maryland institutional animal care and use committee (IACUC), Dr. Lainy Day who had approval from the University of Mississippi IACUC, Dr. Ken Welch Jr. who had approval from the University of California, Riverside IACUC, and Dr. Tim R. Birkhead, who obtained specimens from local hunters in West Woodyates, Dorset, United Kingdom. Other specimens were provided by the Healesville Sanctuary (Healesville, Australia), the Springvale Veterinary Clinic (Springvale, Australia), the Melbourne Zoo (Melbourne, Australia) and the Alberta Institute for Wildlife Conservation (Madden, Canada) staff. In all of these cases, the specimens died from causes unrelated to this project. Some of the songbird specimens in this study were captured in Tippecanoe County, Indiana, USA using mist-nets and live traps by BAM and EF-J. Authorization to capture these birds was obtained from the Indiana Department of Natural Resource and the U.S. Fish and Wildlife Service. Capture and study of all animals did not involve endangered or protected species. The Purdue Animal Care and Use Committee (protocol #1201000567) approved all capturing, handling, and experimental procedures with the birds. Birds were housed indoors in cages (0.9 m60.7 m60.6 m) with 1–3 other individuals of the same species prior to tissue collection. They were kept on a 14:10 hour light:dark cycle and an ambient temperature of approximately 23 C. Food (millet, sunflower seeds and thistle seeds) and water was always provided ad libitum, and supplemented with mealworms (*Tenebrio molitor*) daily. Tissue collection began by euthanizing birds with CO_2_, followed by immediate removal of the eyes for a different study and the head (preserved in 4% paraformaldehyde) for this study.

### Volumetric data Measurements

We measured the volume of SpM, PL, PM, LM, GLv, nBOR, TeO, telencephalon and cerebellum in 100 species (Table S1). For all specimens, the head was immersion-fixed in 4% paraformaldehyde in 0.1 M phosphate buffer (PB). The brain was then extracted, weighed, cryoprotected in 30% sucrose in phosphate buffer and sectioned in the coronal plane on a freezing stage microtome at a thickness of 40 μm. Sections were collected in 0.1 M PB, mounted onto gelatinized slides, stained with thionin and coverslipped. Photomicrographs of every second or every fourth section were taken throughout the rostrocaudal extent of each nucleus using a Retiga EXi FAST Cooled mono 12-bit camera (Qimaging, Burnaby, BC, Canada) and OPENLAB Imaging system (Improvision, Lexington, MA, USA) attached to a compound light microscope (Leica DMRE, Richmond Hill, ON, Canada). For some brains, images of full sections were obtained with a digital slide scanner (Leica SCN400, Richmond Hill, ON, Canada) with a 20X objective. Measurements of all the nuclei were taken directly from these photos with ImageJ (NIH, Bethesda, MD, USA; http://rsb.info.nih.gov/ij/) and volumes were calculated by multiplying the area in each section by the thickness of the section (40 μm) and the sampling interval.

### Statistical analyses

To examine variations in the relative sizes of brain regions, we plotted the log_10_-transformed volume of each brain region against the log_10_- transformed brain volume minus the volume of each specific region. Allometric equations were calculated with least squares linear regressions using phylogenetic generalized least squares (PGLS) to account for phylogenetic relatedness^79^. PGLS allows the covariance matrix to be modified to accommodate the degree to which trait evolution deviates from Brownian motion, through a measure of phylogenetic correlation, *λ*^80^. Our PGLS analyses and maximum likelihood estimates of λ were performed using the *ape*^81^ and *nlme*^82^ packages in R^83^. For phylogenetic relationships, we sampled 500 trees from the Bird Tree project^84^ with a Hackett *et al*.^85^, backbone. We then obtained a maximum clade credibility tree using the *phangorn* package^86^. Confidence and prediction intervals were calculated using *evomap*^87^. We then ran a phylogenetic analysis of covariance (pANCOVA) to determine whether parrots differed from all other birds in the relative size of SpM, telencephalon and cerebellum. Although this analysis was first performed with the consensus tree, we also ran our PGLS models across all 500 trees to account for phylogenetic uncertainty. Using the consensus tree, we also verified the absence of influential data points by excluding each of them one by one from the data and then comparing the derived parameter estimates and fitted values against those that correspond to the model based on the full data. (The R code can be found in http://www.mpcm-evolution.org/practice/online-practical-material-chapter-6). We found no influential cases strongly affecting the interpretations of the model outcomes.

Given that there could be a correlation between the relative size of SpM, the telencephalon and the cerebellum, we also tested for differences in the relative size of SpM, the telencephalon and cerebellum between parrots and other birds using pANCOVA with the rest of the brain, which here is defined as brain volume minus the volume of the telencephalon, cerebellum and optic tectum. We subtracted the optic tectum as it can account for a large amount of the remaining brain volume after the volume of the telencephalon and cerebellum have been removed, and the size of the optic tectum varies significantly among birds^39^. We then tested for correlation between the relative size of SpM and the pontine nuclei, and that of the telencephalon, the cerebellum and the optic tectum. The later was included as a proxy of variation in the size of the visual system as in birds the relative size of most visual areas covaries with that of TeO^38^. In the case of the pontine nuclei, we included the size of visual nuclei that project to each pontine nuclei, LM and Glv in the case of PM, and TeO and nBOR in the case of PL, as covariate in the full model. We restricted the number of variables to avoid further problems with collinearity and avoid overfitting.

To test for this correlation and correct for allometric effects of brain size on all variables, we use PGLS models with SpM volume as the dependent variable, and brain volume as a covariate, as suggested by several authors^88,89^. A caveat of this method to correct for allometric effects is that there is inevitably a high correlation among variables (Table 1) given the allometric effect of brain size in all areas. In turn this can result in high multicollinearity among variables, which can then result in biased estimates of coefficients and/or unreliable coefficients (see below^29,30^). We tested the levels of collinearity between variables by calculating variance inflation factors (VIF) using the *vif* function from the car package in R^90–93^. VIF values were calculated from linear models identical to the PGLS models but with the phylogenetic component removed, as the meaning of VIF within the context of PGLS framework is not obvious. Given the high level of collinearity^92^, we used brain volume minus the volume of the telencephalon, cerebellum and optic tectum as a covariate (remaining brain volume, RBV). This reduced the collinearity between variables (see Table 1). Although these values are above the typical recommended threshold of 10^92,93^, this is not to be taken as an absolute value, as the effects of including collinear variables in the estimation of coefficients and estimability of the model can be assessed (see below). The possible effects of multicollinearity on linear models is widely recognized^29,94^, but how to deal with multicollinearity is unclear, particularly in the context of comparative methods and allometry, and few if any viable options exist. A common way to deal with collinearity in studies that deal with allometry has been to use of phylogenetically corrected residuals against a size variable^95^, but this method can produce biased estimates, particularly in the presence of collinearity^88,89^. Another commonly proposed fix is to remove highly collinear or redundant variables, but in this case all variables are needed to test the proposed hypothesis. Further this method also produces biased estimates given the missing information^28,29^. Several other methods have been proposed to deal with collinearity (reviewed in^94,96^) but these either produce, at best, equally biased estimates (see^28^ for a detailed analysis) and/or have not been developed or tested within a phylogenetic context. We therefore followed the recommendation of Mundry^30^ and checked for the stability of our model. The consequences of collinearity are twofold. First, the impact of individual predictors that are collinear with others can potentially become unreliable and uncertain^93,97,98^. This can be seen in increased standard errors of parameter estimates, and therefore, non-significance (i.e., *P* > 0.05) can be mostly a result of collinearity rather than being symptomatic of absence of an effect. Second, models with high collinearity can, potentially, be very unstable, meaning that small changes in the data can lead to changes in the parameter estimates obtained for the collinear predictors. To test the impact of collinearity in our model we compared the estimated coefficient between a full model and reduced models that were missing one or several variables (see results, Table 1). We also used the same function in R mentioned above to compare coefficient estimates in the full model to coefficients estimated after removing each data point one by one (see results). Both analyses show that even in the presence of collinearity our model was not unstable and removing variables or data points did not change our conclusions significantly (see results).

## Electronic supplementary material

Supplementary Information

Dataset1

## References

[1] Emery, N. J. & Clayton, N. S. The Mentality of Crows: Convergent Evolution of Intelligence in Corvids and Apes. *Science* **306** (2004). 10.1126/science.1098410 15591194

[2] Emery NJ (2006). Cognitive ornithology: the evolution of avian intelligence. Philos. Trans. R. Soc. London B Biol. Sci..

[3] Güntürkün O, Bugnyar T (2016). Cognition without Cortex. Trends Cogn. Sci..

[4] Byrne RW, Whiten A (1992). Cognitive Evolution in Primates: Evidence from Tactical Deception. Man.

[5] Barton RA (1996). Neocortex Size and Behavioural Ecology in Primates. Proc. R. Soc. London B Biol. Sci..

[6] Lefebvre L, Whittle P, Lascaris E, Finkelstein A (1997). Feeding innovations and forebrain size in birds. Anim Behav.

[7] Schoenemann PT, Sheehan MJ, Glotzer LD (2005). Prefrontal white matter volume is disproportionately larger in humans than in other primates. Nat. Neurosci..

[8] Lefebvre L, Reader SM, Sol D (2004). Brains, innovations and evolution in birds and primates. Brain. Behav. Evol..

[9] Whiting B, Barton R (2003). The evolution of the cortico-cerebellar complex in primates: anatomical connections predict patterns of correlated evolution. J. Hum. Evol..

[10] Ramnani N (2006). The primate cortico-cerebellar system: anatomy and function. Nat. Rev. Neurosci..

[11] Barton RA (2012). Embodied cognitive evolution and the cerebellum. Philos. Trans. R. Soc. Lond. B. Biol. Sci..

[12] Barton RA, Venditti C (2014). Rapid Evolution of the Cerebellum in Humans and Other Great Apes. Curr. Biol..

[13] Barton Ra, Harvey PH (2000). Mosaic evolution of brain structure in mammals. Nature.

[14] Balsters JHH (2010). Evolution of the cerebellar cortex: The selective expansion of prefrontal-projecting cerebellar lobules. Neuroimage.

[15] Smaers JB, Steele J, Zilles K (2011). Modeling the evolution of cortico-cerebellar systems in primates. Ann. N. Y. Acad. Sci..

[16] Iwaniuk AN, Dean KM, Nelson JE (2005). Interspecific allometry of the brain and brain regions in parrots (psittaciformes): comparisons with other birds and primates. Brain. Behav. Evol..

[17] Boire D, Baron G (1994). Allometric comparison of brain and main brain subdivisions in birds. J. Hirnforsch..

[18] Glickstein, M. In *Handbook of the Cerebellum and Cerebellar Disorders* 469–478, 10.1007/978-94-007-1333-8_21 (Springer Netherlands, 2013).

[19] Brodal A, Kristiansen K, Jansen J (1950). Experimental demonstration of a pontine homologue in birds. J. Comp. Neurol..

[20] Karten HJ, Finger TE (1976). A direct thalamo-cerebellar pathway in pigeon and catfish. Brain Res..

[21] Zeier H, Karten HJ (1971). The archistriatum of the pigeon: Organization of afferent and efferent connections. Brain Res..

[22] Karten HJ, Hodos W, Nauta WJ, Revzin AM (1973). Neural connections of the ‘visual wulst’ of the avian telencephalon. Experimental studies in the piegon (Columba livia) and owl (Speotyto cunicularia). J. Comp. Neurol..

[23] Wild JM, Williams MN (2000). Rostral Wulst in passerine birds. I. Origin, course, and terminations of an avian pyramidal tract. J. Comp. Neurol..

[24] Wylie, D. R., Gutierrez-Ibanez, C. & Iwaniuk, A. N. Visual-Cerebellar Pathways and their Roles in the Control of Avian Flight. *Front*. *Neurosci*. In press, (2017). 10.3389/fnins.2018.00223 PMC5900027 29686605

[25] Ferran JL (2009). Genoarchitectonic profile of developing nuclear groups in the chicken pretectum. J. Comp. Neurol..

[26] Iwaniuk AN, Hurd PL (2005). The evolution of cerebrotypes in birds. Brain. Behav. Evol..

[27] Iwaniuk AN, Heesy CP, Hall MI, Wylie DRW (2008). Relative Wulst volume is correlated with orbit orientation and binocular visual field in birds. J. Comp. Physiol. A..

[28] Smith AC, Koper N, Francis CM, Fahrig L (2009). Confronting collinearity: comparing methods for disentangling the effects of habitat loss and fragmentation. Landsc. Ecol..

[29] Freckleton RP (2011). Dealing with collinearity in behavioural and ecological data: model averaging and the problems of measurement error. Behav. Ecol. Sociobiol..

[30] Mundry, R. In *Modern Phylogenetic Comparative Methods and Their Application in Evolutionary Biology* 131–153, 10.1007/978-3-662-43550-2_6 (Springer Berlin Heidelberg, 2014).

[31] Sayol F, Lefebvre L, Sol D (2016). Relative brain size and its relation with the associative pallium in birds. Brain. Behav. Evol..

[32] Wullimann MF, Meyer DL (1993). Possible multiple evolution of indirect telencephalo-cerebellar pathways in teleosts: Studies in Carassius auratus and Pantodon buchholzi. Cell Tissue Res..

[33] Iwaniuk AN, Hurd PL, Wylie DR (2006). Comparative morphology of the avian cerebellum: I. Degree of foliation. Brain. Behav. Evol..

[34] Arends JJA, Zeigler HP (1991). Organization of the Cerebellum in the Pigeon (CoZumba Zivia): 11. Projections of the Cerebellar Nuclei. J. Comp. Neurol..

[35] Herculano-Houzel S, Collins CE, Wong P, Kaas JH (2007). Cellular scaling rules for primate brains. Proc. Natl. Acad. Sci. USA.

[36] Olkowicz S (2016). Birds have primate-like numbers of neurons in the forebrain. Proc. Natl. Acad. Sci..

[37] Yopak KE (2010). A conserved pattern of brain scaling from sharks to primates. Proc. Natl. Acad. Sci. USA.

[38] Gutiérrez-Ibáñez, C. *et al*. Mosaic and concerted evolution in the visual system of birds. *Plos One* **9** (2014). 10.1371/journal.pone.0090102 PMC3951201 24621573

[39] Iwaniuk AN, Gutierrez-Ibanez C, Pakan JMP, Wylie DR (2010). Allometric scaling of the tectofugal pathway in birds. Brain. Behav. Evol..

[40] Moore, J. M., Devoogd, T. J. & Moore, J. M. Concerted and mosaic evolution of functional modules in songbird brains. **284** (2017). 10.1098/rspb.2017.0469 PMC5443954 28490627

[41] Gutiérrez-Ibáñez C, Iwaniuk AN, Wylie DR (2011). Relative size of auditory pathways in symmetrically and asymmetrically eared owls. Brain. Behav. Evol..

[42] Kaas JH (2004). Evolution of somatosensory and motor cortex in primates. Anat. Rec..

[43] Kaas JH (2012). The evolution of neocortex in primates. Prog. Brain Res..

[44] Shanahan M, Bingman VP, Shimizu T, Wild M, Güntürkün O (2013). Large-scale network organization in the avian forebrain: a connectivity matrix and theoretical analysis. Front. Comput. Neurosci..

[45] Iwaniuk AN, Wylie DRW (2006). The evolution of stereopsis and the Wulst in caprimulgiform birds: A comparative analysis. J. Comp. Physiol. A. Neuroethol. Sens. Neural. Behav. Physiol..

[46] Cunningham SJ (2013). The anatomy of the bill tip of kiwi and associated somatosensory regions of the brain: comparisons with shorebirds. Plos One.

[47] Iwaniuk AN, Clayton DH, Wylie DRW (2006). Echolocation, vocal learning, auditory localization and the relative size of the avian auditory midbrain nucleus (MLd). Behav. Brain Res..

[48] Güntürkün O (2012). The convergent evolution of neural substrates for cognition. Psychol. Res..

[49] Güntürkün O (2005). The avian ‘prefrontal cortex’ and cognition. Current Opinion in Neurobiology.

[50] Ramnani N (2012). Frontal Lobe and Posterior Parietal Contributions to the Cortico-cerebellar System. The Cerebellum.

[51] Wild JM, Farabaugh SM (1996). Organization of afferent and efferent projections of the nucleus basalis prosencephali in a passerine, Taeniopygia guttata. J. Comp. Neurol..

[52] Ito M (2008). Control of mental activities by internal models in the cerebellum. Nat. Rev. Neurosci..

[53] Miller EK, Cohen JD (2001). An Integrative Theory of Prefrontal Cortex Function. Annu. Rev. Neurosci..

[54] Schmahmann JD (2000). The role of the cerebellum in affect and psychosis. J. Neurolinguistics.

[55] Ito M (2006). Cerebellar circuitry as a neuronal machine. Prog. Neurobiol..

[56] Smaers JB (2014). Modeling the evolution of the cerebellum: from macroevolution to function. Prog. Brain Res..

[57] Clarke PG (1977). Some visual and other connections to the cerebellum of the pigeon. J. Comp. Neurol..

[58] Wild JM (1992). Direct and indirect ‘cortico’-rubral and rubro-cerebellar cortical projections in the pigeon. J. Comp. Neurol..

[59] Iwaniuk, A., Hurd, P. & Wylie, D. Comparative morphology of the avian cerebellum: II. Size of folia. *Brain*. *Behav*. *Evol* (2006). 10.1159/000096987 17108672

[60] Herculano-Houzel S (2010). Coordinated scaling of cortical and cerebellar numbers of neurons. Front. Neuroanat..

[61] Homberger, D. G. Functional morphology and evolution of the feeding apparatus in parrots, with special reference to the Pesquet’s Parrot, Psittrichas fulgidus (lesson). In *Conservation of New World parrots: Proceedings of the ICBP Parrot Working Group Meeting*, *St*. *Lucia* 471 (1980).

[62] Homberger, D. G. The comparative biomechanics of a prey-predator relationship: the adaptive morphologies of the feeding apparatus of Australian Black-Cockatoos and their foods as a basis for the reconstruction of the evolutionary history of the Psittaciformes. *Vertebr*. *Biomech*. *Evol*. 203–228 (2003).

[63] Zweers, G. A., Berkhoudt, H. & Vanden Berge, J. C. In 241–279, 10.1007/978-3-642-57906-6_9 (Springer, Berlin, Heidelberg, 1994).

[64] Proville RD (2014). Cerebellum involvement in cortical sensorimotor circuits for the control of voluntary movements. Nat. Neurosci..

[65] Blakemore S-J, Sirigu A (2003). Action prediction in the cerebellum and in the parietal lobe. Exp. Brain Res..

[66] Miceli D, Repérant J, Villalobos J, Dionne L (1987). Extratelencephalic projections of the avian visual Wulst. A quantitative autoradiographic study in the pigeon Columbia livia. J. Hirnforsch..

[67] Medina L, Reiner A (2000). Do birds possess homologues of mammalian primary visual, somatosensory and motor cortices?. Trends Neurosci..

[68] Kröner S, Güntürkün O (1999). Afferent and efferent connections of the caudolateral neostriatum in the pigeon (Columba uvia): A retro- and anterograde pathway tracing study. J. Comp. Neurol..

[69] Atoji Y, Wild JM (2009). Afferent and efferent projections of the central caudal nidopallium in the pigeon (*Columba livia*). J. Comp. Neurol..

[70] Tamada T, Miyauchi S, Imamizu H, Yoshioka T, Kawato M (1999). Cerebro‐cerebellar functional connectivity revealed by the laterality index in tool‐use learning. Neuroreport.

[71] Symes CT, Perrin MR (2003). Feeding biology of the Greyheaded Parrot, Poicephalus fuscicollis suahelicus (Reichenow), in Northern Province, South Africa. EMU.

[72] Morales Picard A (2017). Diffusion of novel foraging behaviour in Amazon parrots through social learning. Anim. Cogn..

[73] Schuck-Paim C, Borsari A, Ottoni EB (2009). Means to an end: Neotropical parrots manage to pull strings to meet their goals. Anim. Cogn..

[74] Borsari A, Ottoni EB (2005). Preliminary observations of tool use in captive hyacinth macaws (Anodorhynchus hyacinthinus). Anim. Cogn..

[75] Bond A, Diamond J (2003). A Comparative Analysis of Social Play in Birds. Behaviour.

[76] Kerney, M., Smaers, J. B., Schoenemann, P. T. & Dunn, J. C. The coevolution of play and the cortico-cerebellar system in primates. *Primates* 1–7, 10.1007/s10329-017-0615-x (2017). 10.1007/s10329-017-0615-x PMC5622916 28620843

[77] Middleton FA, Strick PL (2001). Cerebellar Projections to the Prefrontal Cortex of the Primate. J. Neurosci..

[78] Medina L, Veenman CL, Reiner A (1997). Evidence for a possible avian dorsal thalamic region comparable to the mammalian ventral anterior, ventral lateral, and oral ventroposterolateral nuclei. J. Comp. Neurol..

[79] Garland T, Ives AR (2000). Using the past to predict the present: confidence intervals for regression equations in phylogenetic comparative methods. Am. Nat..

[80] Pagel M (1999). Inferring the historical patterns of biological evolution. Nature.

[81] Paradis E, Claude J, Strimmer K (2004). A {PE}: analyses of phylogenetics and evolution in {R} language. Bioinformatics.

[82] Pinheiro, J., Bates, D., DebRoy, S., Sarkar, D. & R Core Team. {nlme}: Linear and Nonlinear Mixed Effects Models (2017).

[83] R Core Team. R: A Language and Environment for Statistical Computing (2016).

[84] Jetz, W., Thomas, G. H., Joy, J. B., Hartmann, K. & Mooers, A. O. The global diversity of birds in space and time. *Nature* **491** (2012). 10.1038/nature11631 23123857

[85] Hackett SJ (2008). A phylogenomic study of birds reveals their evolutionary history. Science.

[86] Schliep K (2011). P. phangorn: phylogenetic analysis in R. Bioinformatics.

[87] Smaers JB, Rohlf FJ (2016). Testing species deviation from allometric predictions using the phylogenetic regression. Evolution.

[88] Freckleton R (2002). On the misuse of residuals in ecology: regression of residuals vs. multiple regression. J. Anim. Ecol..

[89] Darlington RB, Smulders TV (2001). Problems with residual analysis. Anim. Behav..

[90] Fox, J. & Weisberg, S. An R Companion to Applied Regression, 2nd edn. An R package ‘car’, version 1.2–16 from August 2012 (2011).

[91] Team, R. C. R. A Language and Environment for Statistical Computing (2015).

[92] O’Brien RM (2007). A caution regarding rules of thumb for variance inflation factors. Qual. Quant..

[93] Quinn, G. P. & Keough, M. J. *Experimental design and data analysis for biologists*. (Cambridge University Press, 2002).

[94] Dormann CF (2013). Collinearity: a review of methods to deal with it and a simulation study evaluating their performance. Ecography (Cop.)..

[95] Revell LJ (2009). Size-correction and principal components for interspecific comparative studies. Evolution.

[96] Kiers HAL, Smilde AK (2007). A comparison of various methods for multivariate regression with highly collinear variables. Stat. Methods Appl..

[97] Field, A. Discovering statistics using SPSS 2nd edn Sage Publications (2005).

[98] Zuur AF, Ieno EN, Elphick CS (2010). A protocol for data exploration to avoid common statistical problems. Methods Ecol. Evol..

