# Supplementary material for: Parrots have evolved a primate-like telencephalic-midbrain-cerebellar circuit
Source: Sci Rep. 2018 Jul 2;8:9960. doi: 10.1038/s41598-018-28301-4 (PMC6028647; doi:10.1038/s41598-018-28301-4)
Supplement: Supplementary file 1 — Supplementary Information [file 41598_2018_28301_MOESM1_ESM.pdf]

Parrots have evolved a primate-like telencephalic-midbrain-cerebellar circuit

Cristian Gutierrez-Ibanez*[1], Andrew N. Iwaniuk[2], Douglas R. Wylie[1]

Supplementary Information

Supplementary Materials and Methods

Borders of nuclei

We based the borders of SpM and the pontine nuclei on several brain atlases and other publications. SpM is readily recognizable in Nissl stained sections as a densely packed group of cells medial to the nucleus pretectalis (Fig S2 A-B). SpM has two generally recognized subdivisions, a lateral and a medial (Fig S2B). Ventrally, it borders with the occipitalomesencephalic tract (OM). Ferran et al. (26), based on immunohistochemistry of several markers, suggest that the rostral most part of what has been recognized as SpM in some publication (24) is a distinct nucleus, the dorsofrontal nucleus. This nucleus is continuous with SpM (26), and projects to the cerebellum in zebra finches (24), hummingbirds and pigeons (personal observations), so it was included in our measurement of SpM.

The pontine nuclei lie at the base of the brainstem and extends from levels anterior to the trochlear nucleus until the root of the VI nerve. PM is generally round with big, darkly stained cells and lies just lateral to the raphe nucleus in the midline. Caudally it extends to the level of the IV nerve root, which act at its lateral border (Fig S2). PL is usually found more anterior than PM. Its anterior portion lies dorsal and lateral in the brainstem and start just posterior to the nucleus semilunaris (Slu). At more posterior level its lies more ventral and medial. At this level, the ventral part of lateral lemniscus nucleus

(LLV) can be recognized just dorsal to the lateral most portion of PL. At very posterior levels the root of the VI nerves becomes its medial most border.

Supplementary Figure captions

Figure S1. Distribution of P-values for differences in intercept between parrots and all other species in PGLS models with SpM volume as the dependent variable and Brain volume – SpM volume as the independent variable, run with 500 different phylogenetic trees to account for phylogenetic uncertainty All bird phylogenies were acquired from www.birdtree.org. Lambda was set at its maximum likelihood value each time. The red line shows the average P-value, p = 0.0193.

Figure S2. Location, borders and cytoarchitecture of the medial spiriform (SpM) nucleus and the pontine nuclei. Photomicrographs showing the location and borders of SpM and the pontine nuclei in different species of birds. **A** shows a microphotograph of a Nissl stained coronal sections through the posterior part of SpM in a parrot (Psittaciformes), the long-billed corella (*Cacatua tenuirostris*). **B** shows the same in a Gruiforme, the America coot (*Fulica amaricana*). **C** and **D** show the borders of the medial (PM) and lateral (PL) pontine in a Procellariiform the short-tailed shearwater or slender-billed shearwater (*Ardenna tenuirostris)* and an owl (Strigiformes), the great grey owl *(Strix nebulosa)*. PT= nucleus pretectalis.

Table S2. Results of phylogenetic least-squares linear regression (PGLS) performed on the log-transformed volume the magnocellular and parvocellular portions of nucleus spiriformis medialis (SpM), lateral and medial pontine nucleus(PM and PL), the ventral part of the geniculate nucleus (Glv), the nucleus of the basal optic root (nBOR), the nucleus *lentiformis mesencephalic (LM), the optic tectum (TeO), the telencephalon (Tel) and the cerebellum (Cb)* against the log-transformed brain volume minus the volume of the respective nuclei.

	log brain-region volume			
	**Intercept ±SE**	**Slope ±SE**	**Adj R^2**	**Lambda** (95.0% CI)
SpM	-3.08±0.16	0.85±0.043	0.797	0.943 (0.85, 0.986)
PM	-2.65±0.19	0.71±0.051	0.684	0.977* (0.814, NA)
PL	-2.54±0.18	0.722±0.047	0.729	0.844 (0.511, 0.99)
Glv	-1.80±0.18	0.587±0.047	0.611	0.804 (0.601, 0.924)
nBOR	-2.36±**0**.20	0.709±0.05	0.659	0.899 (0.738, 0.981)
LM	-2.11±0.139	0.692±0.037	0.7728	0.585 (0.315, 0.814)
TEO	-0.140±0.16	0.648±0.044	0.685	0.902 (0.743, 0.971)
Tel	0.142±0.19	1.010±0.055	0.7923	0.953* (0.798, NA)
Cb	-0.402±0.15	0.86±0.04	0.8328	0.8328 (0.581, 0.948)

*not significantly different form 1.

Table S3. Results of phylogenetic Analysis of covariance (pANCOVA), comparing the relative size of the nucleus spiriformis medialis (SpM) and the lateral and medial pontine nucleus (PM and PL) between different orders of birds and all other birds. Analysis where performed with the consensus tree (see methods)

	Parrots		Pelecaniformes		Waterfowl		Owls		Passeriforms	
	F	p	F	p	F	p	F	p	F	p
SpM	5.04	0.027	2.73	0.10	2.25	0.14	0.62	0.43	0.51	0.47
PM	0.44	0.508	0.14	0.71	0.38	0.54	0.82	0.36	0.19	0.66
PL	0.93,	0.34	0.27	0.60	3.46	0.066	0.007	0.93	0.81	0.37

Table S4. Results of PGLS models with PM as the dependent variable. RBV is the remaining brain volume, brain volume minus telencephalon (Tel), cerebellum (Cb) and optic tectum (TeO) volume.

model	ʎ	AIC	variable	VIF*	β	S.E	t	P value
Full	1	-130.96	RBV	5.92	-0.013	0.064	-0.209	0.835
			TeL	12.66	0.315	0.121	2.605	0.011
			Cb	13.95	0.227	0.106	2.141	0.036
			Glv	7.54	0.342	0.103	3.311	0.001
			LM	15.46	0.057	0.074	0.764	0.447
Reduced 2	1	-133.92	RBV	5.71	-0.006	0.063	-0.098	0.923
			TeL	12.31	0.324	0.120	2.696	0.009
			Cb	12.24	0.198	0.099	2.006	0.049
			Glv	5.00	0.380	0.090	4.199	0.000
Reduced 3	1	-114.67	RBV	5.82	-0.010	0.069	-0.147	0.884
			TeL	12.64	0.372	0.128	2.904	0.005
			Cb	13.94	0.350	0.106	3.302	0.002
			LM	10.25	0.175	0.070	2.503	0.015
Reduced 4	1	-118.98	RBV	5.17	0.020	0.070	0.290	0.772
			Tel	12.25	0.430	0.130	3.304	0.001
			Cb	11.01	0.290	0.107	2.713	0.008
Reduced 5	1	-114.03	RBV	4.92	-0.018	0.072	-0.254	0.800
			TeL	4.92	0.765	0.044	17.355	0.000
Reduced 6	0.97	-87.14	RBV	4.42	0.141	0.066	2.128	0.037
			Cb	4.42	0.653	0.076	8.627	0.000

Analysis where performed with the consensus tree (see methods).

Table S5. Results of PGLS models with PL as the dependent variable. RBV is the remaining brain volume, brain volume minus telencephalon (Tel), cerebellum (Cb) and optic tectum (TeO) volume. Analysis where performed with the consensus tree (see methods).

model	ʎ	AIC	variable	VIF*	β	S.E	t	P value
Full	0.52	-82.08	RBV	5.92	0.040	0.077	0.521	0.604
			TeL	12.73	0.502	0.135	3.708	0.000
			Cb	16.55	0.329	0.147	2.243	0.028
			TeO	7.96	-0.069	0.127	-0.541	0.590
			nBOR	5.88	-0.103	0.103	-0.995	0.323
			LM	11.02	0.033	0.134	0.245	0.808
Reduced 2	0.57	-83.04	RBV	5.86	0.043	0.077	0.564	0.574
			TEL	12.63	0.488	0.136	3.598	0.001
			CB	16.14	0.298	0.147	2.027	0.046
			TeO	6.71	-0.102	0.122	-0.835	0.406
			LM	10.83	0.014	0.133	0.106	0.916
Reduced 3	0.57	-85.03	RBV	5.32	0.045	0.075	0.600	0.551
			TeL	12.27	0.489	0.134	3.644	0.001
			Cb	14.4	0.303	0.138	2.200	0.031
			TeO	6.34	-0.098	0.117	-0.845	0.401
Reduced 4	0.61	-86.29	RBV	5.17	0.038	0.075	0.507	0.614
			Tel	12.25	0.475	0.133	3.570	0.001
			Cb	11.01	0.256	0.130	1.963	0.053
Reduced 5	0.77	-85.31	RBV	4.92	0.016	0.077	0.205	0.838
			TeL	4.92	0.707	0.077	9.217	0.000
Reduced 6	0.55	-75.99	RBV	4.42	0.152	0.073	2.078	0.041
			Cb	4.42	0.648	0.076	8.552	0.000

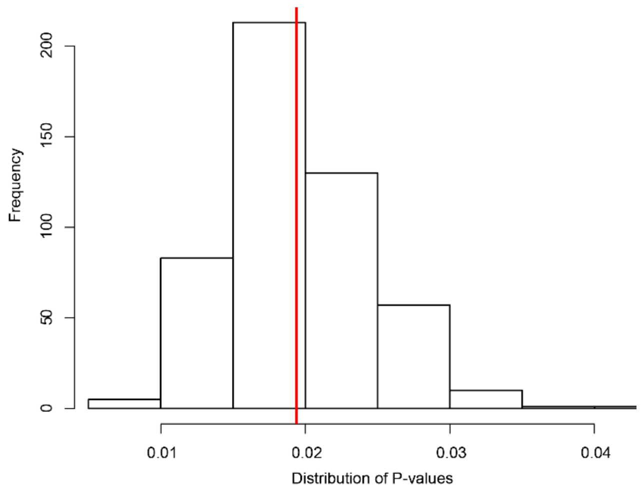

Figure S1

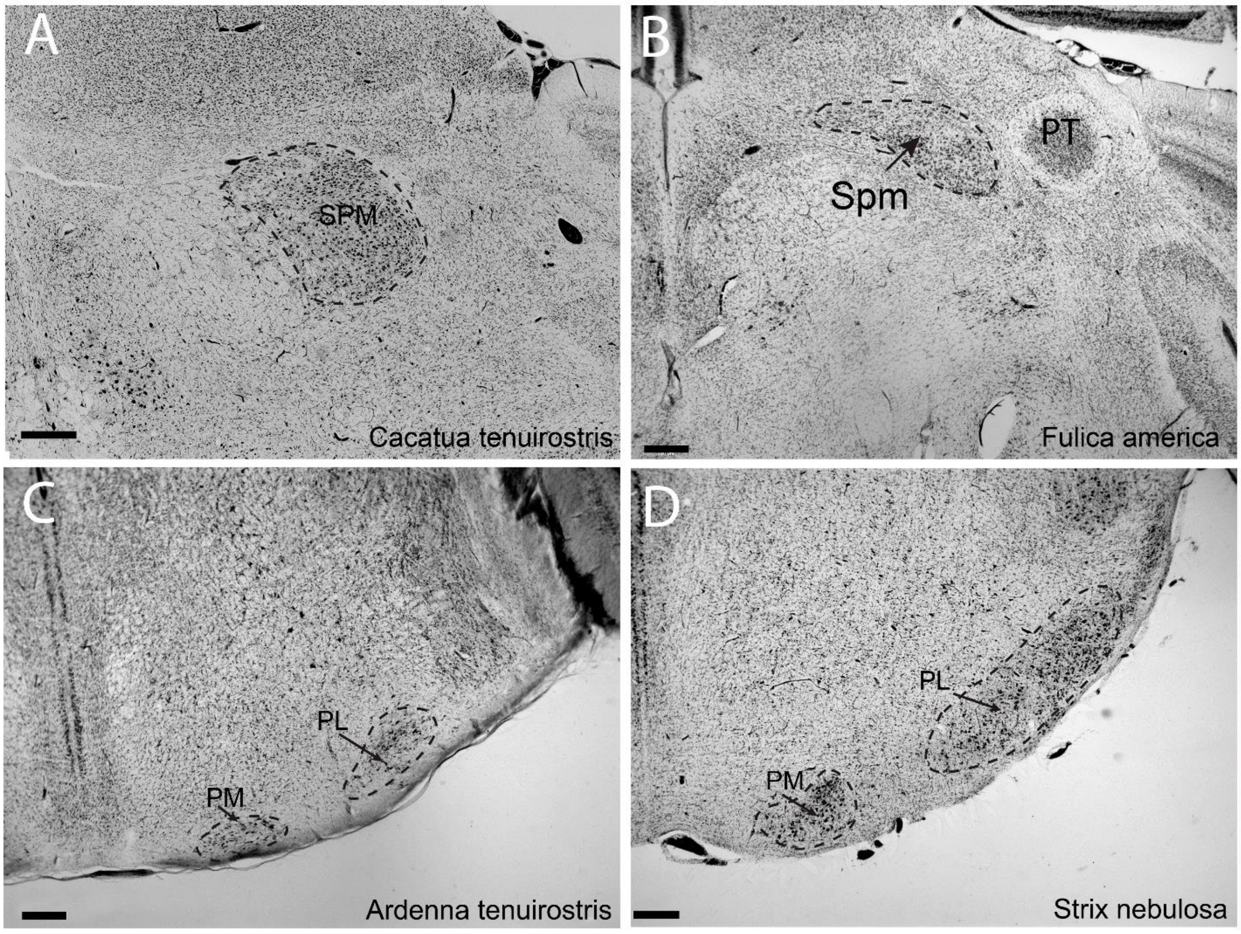

Figure S2
